# Changes in bacterioplankton community structure during early lake ontogeny resulting from the retreat of the Greenland Ice Sheet

**DOI:** 10.1038/ismej.2017.191

**Published:** 2017-10-31

**Authors:** Hannes Peter, Erik Jeppesen, Luc De Meester, Ruben Sommaruga

**Affiliations:** 1 Institute of Ecology, Lake and Glacier Ecology Research Group, University of Innsbruck, Innsbruck, Austria; 2 Aarhus University, Department of Bioscience, Silkeborg, Denmark; 3 Sino-Danish Centre for Education and Research (SDC), University of Chinese Academy of Sciences, Beijing, China; 4 Laboratory for Aquatic Ecology, Evolution and Conservation, KU Leuven, Leuven, Belgium

## Abstract

Retreating glaciers and ice sheets are among the clearest signs of global climate change. One consequence of glacier retreat is the formation of new meltwater-lakes in previously ice-covered terrain. These lakes provide unique opportunities to understand patterns in community organization during early lake ontogeny. Here, we analyzed the bacterial community structure and diversity in six lakes recently formed by the retreat of the Greenland Ice Sheet (GrIS). The lakes represented a turbidity gradient depending on their past and present connectivity to the GrIS meltwaters. Bulk (16S rRNA genes) and putatively active (16S rRNA) fractions of the bacterioplankton communities were structured by changes in environmental conditions associated to the turbidity gradient. Differences in community structure among lakes were attributed to both, rare and abundant community members. Further, positive co-occurrence relationships among phylogenetically closely related community members dominate in these lakes. Our results show that environmental conditions along the turbidity gradient structure bacterial community composition, which shifts during lake ontogeny. Rare taxa contribute to these shifts, suggesting that the rare biosphere has an important ecological role during early lakes ontogeny. Members of the rare biosphere may be adapted to the transient niches in these nutrient poor lakes. The directionality and phylogenetic structure of co-occurrence relationships indicate that competitive interactions among closely related taxa may be important in the most turbid lakes.

## Introduction

Climate change causes a massive, and on a millennial timescale unprecedented retreat of glaciers and ice sheets. Among the most-sensitive and most-affected areas is the Greenland Ice Sheet (GrIS) (Hanna *et al.*, 2013). Surface ice mass balances for the GrIS estimated an average mass loss of 186.4 Gt per year between 2003 and 2010, which is ca. 2.5 times higher than in the preceding century (Kjeldsen *et al.*, 2015). In 2012, the melting of GrIS occurred for the first time in this century during 120 days and surface ice melting comprised up to 98.6% of the GrIS total area (Nghiem *et al.*, 2012).

Although a substantial fraction of the GrIS meltwaters enters the ocean directly (Bamber *et al.*, 2012), where land topology allows, new lakes have been (and will be) created (Carrivick and Tweed, 2013). Many of the newly formed lakes may dry out or are unstable and drain rapidly when moraines, landslide debris accumulations or ice dams break (Carrivick and Tweed, 2013). However, many lakes persist and provide an exceptional opportunity to gain insight into the dynamics of community composition and structure during early lake ontogeny (Haileselasie *et al.*, 2016). The planktonic biota in such newly created lakes is mainly composed of microbes (Sommaruga, 2015), and keystone groups such as heterotrophic flagellates or *Daphnia* are absent or reduced in abundance (Koenings *et al.,* 1990; Sommaruga and Kandolf, 2014; Peter and Sommaruga, 2016). One environmental condition that largely explains this particular food web structure is the high concentration of suspended mineral particles, which is responsible for the turbidity of these lakes (Sommaruga, 2015; Peter and Sommaruga, 2016). Turbidity also restricts the penetration of light available for primary production (Rose *et al.*, 2014). In addition, the GrIS meltwaters supply nutrients such as phosphorus and nitrogen (Hawkings *et al.*, 2016), bioavailable iron (Bhatia *et al.*, 2013b) and labile organic carbon (Bhatia *et al.*, 2013a; Lawson *et al.*, 2014), favoring the growth of heterotrophic microbes.

Here, we analyzed the structure and diversity patterns of bulk (16S rRNA genes, hereafter, termed 16S rDNA) and putatively active (the transcript of the 16S rRNA genes, termed 16S rRNA) fractions of bacterioplankton communities, as well as key environmental factors of six lakes that have been created by the retreat of the GrIS. Despite the fact that all lakes are young (<40 years) and close to the ice margin (<2 km distant), they have different degrees of connectivity with the GrIS runoff, which is reflected by a gradient in turbidity. Assuming a unidirectional retreat of the GrIS, the turbidity gradient also reflects somehow a gradient in lake ontogenetic age, with the most turbid lakes being most recently formed.

First, we hypothesized that the rare biosphere (that is, the long tail on species-abundance curves) accumulates during early lake ontogeny, resulting in an increased contribution of rare taxa in lakes of lower turbidity (that is, disconnected from the GrIS for longer time). Rare taxa have been categorized to be either persistently or conditionally rare (Shade *et al.*, 2014; Lynch and Neufeld, 2015; Newton and Shade, 2016). Whereas persistently rare taxa may be not as readily dispersed as more abundant members of the regional species pool, conditionally rare taxa may require time to accumulate during phases of favorable and unfavorable conditions. Although rare taxa have important roles as reservoirs of genetic and functional diversity and rarity may be a successful adaptation to low resource availability (Lynch and Neufeld, 2015; Newton and Shade, 2016), inactive taxa could substantially contribute to the rare biosphere. Based on the notion that active community members turn over quickly in response to changing environmental conditions, we further hypothesized that the contribution of rare taxa to beta-diversity is more pronounced for bulk than for active fractions of the communities along the environmental gradient.

Moreover, we investigated the phylogenetic structure of co-occurring taxa in these recently formed lakes. The truncated food web, the young age and the close spatial proximity of these lakes offer ideal circumstances to address such questions. Although different evolutionary and ecological forces may contribute to patterns in phylogenetic relatedness among co-occurring taxa, environmental filtering and species interactions have been proposed to result in phylogenetic clustering or overdispersion, respectively (Webb *et al.,* 2002; Mouquet *et al.,* 2012). Phylogenetic clustering reflects smaller distances towards the tips of a phylogenetic tree, whereas overdispersion reflects a greater phylogenetic dispersion. A clustered phylogenetic distribution may indicate shared environmental preferences of closely related taxa. Competition among closely related taxa with similar niche preferences, in contrast, may lead to phylogenetic overdispersion. We first inferred positive (co-presence) and negative (mutual exclusion) co-occurrence relationships (Faust *et al.,* 2012) of bacterial taxa along the turbidity gradient and contrasted the phylogenetic relationships of co-occurring taxa to the bulk bacterioplankton communities. We hypothesized that the nutrient poor environment of these newly formed lakes imposes strong environmental filters on the communities and that this is reflected in the phylogenetic distribution.

## Materials and methods

### Study area and sampling

A set of six lakes formed by the retreat of the GrIS in the Jakobshavn Isbræ region of western Greenland (69°06'16‘ N, 49°44'43’ W) were sampled during August 2012 (Supplementary Figure S1). Analysis of Landsat images (July-September) and of an aerial orthoimages revealed that in 1972, this area was still covered by GrIS, so all lakes were <40 years old at the time of sampling (Supplementary Figure S2). Considering the existence of a defined shoreline as criterion to define a system, the GrIS meltwaters created by ca. 1985 Lake IL2 (turbidity_2012_: 11.1 NTU) and by 1994 Lake IL7 (turbidity_2012_: 0.82 NTU) started to be created as a side system from a much larger lake that later drained, so that by ca. 1998 it was a separated waterbody. Lake IL16 (turbidity_2012_: 5.46 NTU) is very small and difficult to identify in images, but formed probably by a secondary moraine after IL2 was created. All other lakes were created between 2002 and 2008. Here, we use the turbidity gradient (ranging from 0.82–64.1 NTU) as a proxy for the connectivity to GrIS. It is clear that this is a dynamic landscape and that the ontogenetic age and turbidity may not be linearly related.

This remote area was reached by helicopter from Illulisat (thus ‘IL’) and a basecamp was established for 14 days. The lakes were fish-free and had no (IL9 and IL15) or sparse populations of *Daphnia* (<0.6 ind. l^−1^). Three replicated composite water samples of 10 l were taken above the deepest point in each lake. Equal volumes of water from different depths were pooled along the water column (number of depths sampled ranged from 4–11). However, in L15, only the uppermost 20 m could be sampled. From the replicated composite water samples, we sampled immediately for bacterial community analyses, turbidity measurements, dissolved organic carbon (DOC) concentration and optical characterization, whereas all other analyses (nutrients, chlorophyll-a) were analyzed from just one of the replicates due to logistical constraints.

### Physico-chemical parameters

*In situ* measurements of water temperature, conductivity and pH were done with an YSI multiparameter sonde (model 6600 V2). Nephelometric turbidity of the composite water samples was measured (three times) using a portable instrument (Turb 430T, WTW, Germany) measuring 90° scattered ‘white’ light (Peter and Sommaruga, 2017). Total phosphorus was determined as molybdate reactive phosphorus (Murphy and Riley, 1962) following persulphate digestion (Koroleff, 1970) and total nitrogen as nitrite+nitrate after potassium persulphate digestion (Solórzano and Sharp, 1980). Soluble reactive phosphorus and nitrite+nitrate in the dissolved fraction were below detection levels, with exception of the two systems directly receiving GrIS meltwaters (IL9 and IL15). Chlorophyll-a was determined spectrophotometrically after ethanol extraction (Jespersen and Christoffersen, 1987). Samples for DOC were filtered through two pre-combusted (450 °C for 2 h) glass fiber GF/F filters (Whatman), and the filtrate was collected in pre-treated (3 weeks in 0.2 n HCl and rinsed several times with Milli-Q water) polypropylene centrifuge tubes (50 ml). Three blanks were prepared by filling the tubes with filtered (as above) Milli-Q water. The filtrate was acidified with HCl to pH 2 and the samples were kept frozen until analysis in a Shimadzu analyzer (TOC-Vc series). Before analysis, the samples were homogenized for 30 s with a tip ultrasonic probe. Three to five subsamples were analyzed for each sample and for one sample of consensus reference material for DOC (batch 5 FS-2005: 0.57 mg; provided by RSMAS/MAC, University of Miami). Results differed from the consensus reference material by <5% and the coefficient of variation among subsamples was <2%. From the same filtrate as for DOC (but without acidification), spectral characteristics of the chromophoric dissolved organic matter were measured in a double-beam spectrophotometer (Hitachi 2500) with a 10-cm cuvette. We calculated the ratio of the slopes (*S*_R_) of log-transformed absorption over two wavelength regions (275–295 and 350–400 nm) as a proxy (inversely related) of the dominant molecular weight of DOM (Helms *et al.*, 2008). Spectrofluorometric measurements (McKnight *et al.*, 2001) (excitation: 240–450 nm in 10 nm increments, emission: 300–560 in 2 nm increments, 0.25s integration time) were made with a Fluoromax-4 fluorometer (Horiba, Jobin Yvon). Instrument-specific corrections (that is, S1c/R1c which subtracts blanks, removes dark noise and corrects for inhomogeneity in the detector response) were applied during spectral acquisition. Excitation-emission matrices (EEMs) were processed using the eemR package in R. Specifically, Milli-Q water blanks were subtracted from the samples, 1st and 2nd order Rayleigh and Raman scattering areas were removed and the inner-filter effect was corrected using the absorbance spectra obtained for chromophoric dissolved organic matter characterization. Finally, fluorescence intensities were normalized to Raman Units based on the area of the Raman peak measured from the Milli-Q blank. The relative distribution of Coble peaks b and t, which represent protein-like compounds and Coble peaks a, c and m, which represent humic-like components (Coble, 1996), were identified.

### Bacterial community analysis

We used next-generation amplicon sequencing of the 16S rDNA and 16S rRNA to describe the bacterioplankton communities. While 16S rDNA is commonly used to assess the genetic diversity of complex microbial assemblages, the ribosomal RNA has been used to identify active fractions of environmental communities (for example, Logue and Lindström, 2010; Besemer *et al.*, 2012; Wilhelm *et al.*, 2014; see Blazewicz *et al.*, 2013 for a review). However, depending on life history, RNA concentrations and growth rates may not be linearly related and may differ for different taxa. Dormant and metabolically inactive cells may also contain large amounts of RNA. Therefore, the dynamics of the 16S rRNA diversity reported here should be regarded as the result of past and current microbial activities (Blazewicz *et al.*, 2013).

Samples were filtered onto a 0.2 μm polyethersulfone filter (GPWP, Merck Millipore, Ireland) until clogging occurred. In cases where the volume filtered was small (<250 ml) owing to high turbidity, two to three filters were prepared for each replicate. Next, the filters were placed in a cryovial and left overnight at low temperature (6 °C) with RNAlater stabilization reagent (Qiagen, Hilden, Germany), stored in liquid nitrogen during transport, and finally stored at −80 °C.

Nucleic acids (DNA and RNA) were extracted from the filters using the PowerWater DNA extraction kit (Mobio, Carlsbad, CA, USA). A similar kit has been shown to yield high DNA and RNA concentrations (Besemer *et al.*, 2012). Following a previously published protocol for transcription of RNA to complementary DNA (Logue and Lindström, 2010), genomic DNA was first removed from an aliquot using DNaseI (Invitrogen, Carlsbad, CA, USA), followed by reverse transcription using random oligonucleotide primer and RevertAid H Minus transcriptase (ThermoFisher, Waltham, MA, USA). PCR using the product prior to the reverse transcription and gel electrophoresis served as negative controls. Although these controls were negative, we are aware that RNA extraction and transcription may not be quantitative (Blazewicz *et al.,* 2013) and we cannot fully exclude the possibility that the co-extraction of rRNA and rDNA may have caused cross-contamination of the rRNA fraction.

Bacterial community composition was analyzed by sequencing of the V4 region of the 16S rRNA gene on the 454 GS FLX platform with Titanium chemistry (Roche, Switzerland). Triplicated PCR reactions using the barcode-primer combinations described in Fierer *et al.* (2008), purification, quantification and equimolar mixing of the samples prior to sequencing were done by EnGencore (Greenville, SC, USA). In brief, PCR reactions were prepared with the following reagents: 10 μl 5-Prime Hot Master Mix (5Prime; Gaithersburg, MD, USA), 30 μm of each forward and reverse primer, and 15 ng of template DNA. Thermal cycling conditions were set to an initial denaturation at 94 °C for 3 min followed by 30 cycles of denaturation at 94 °C for 1 min, annealing at 50 °C for 30 s, and extension at 72 °C for 2 min, with a final extension of 10 min at 72 °C. Triplicate PCRs from each sample were combined and purified using the QIAquick PCR purification kit (Qiagen), followed by additional purification with AMPure beads (Beckman Coulter, Brea, CA, USA). The PCR products were quantified with PicoGreen (Invitrogen) and pooled in equimolar ratios.

### Data analysis

Bioinformatic analyses were conducted using mothur following the Standard Operational Protocol (Schloss *et al.*, 2011), including PyroNoise, which reduces the sequencing error rate by correcting the original flowgram data. Sequences were aligned against the SILVA reference database (v128) and taxonomically assigned using mothur’s naive Bayesian classifier. Sequences identified as Eukaryota, Archaea, mitochondria or unknown phyla were removed. Chimeric sequences were removed using uchime (Edgar *et al.*, 2011). Pairwise sequence distances were calculated treating gaps of any length as single insertions and sequences were clustered into operational taxonomic units (OTUs) at the 97% similarity level. After denoising, 80197 high quality sequences (12.9% of the initial reads) remained in the rDNA and 43942 (19.8%) sequences remained in the rRNA fraction.

16S rRNA genes classified as chloroplasts in the SILVA database were aligned against the PhytoRef database (Decelle *et al.*, 2015) and classified after removal of potential chimeric sequences. The PhytoRef database contains 6490 plastidial 16S rDNA reference sequences spanning all major photosynthetic lineages, however is dominated by marine microalgae. Although this is currently the only publicly available resource to assess the relative abundance and diversity of photosynthetic eukaryotes, several limitations should be kept in mind. First, the plastidial 16S rDNA provides less taxonomic resolution than for example the large subunit of the ribulose 1,5-bisphosphate carboxylase genes for plants. The relative counts obtained by querying PhytoRef may also be affected by the number of plastids different species harbor or, probably to a lesser extent, by plastidial 16S rDNA gene copy numbers (Decelle *et al.*, 2015).

Statistical analyses and figures were prepared using the statistical environment R and the packages vegan (Oksanen *et al.*, 2013) and picante (Kembel *et al.*, 2010). Data sets for multivariate statistical analyses, diversity estimates and co-occurrence network creation were rarefied to 1863 and 1052 sequences for rDNA and rRNA, respectively, using the ’rrarefy’ function in vegan. Bootstrap OTU richness, Chao-1 estimates, the inverse Simpson index (that is, a measure of evenness) and phylogenetic diversity (Faith’s PD) were calculated to assess different aspects of biodiversity. The phylogenetic tree used for PD was calculated using FastTree vers. 2.1.7, applying the generalized time-reversible model. To assess the contribution of rare taxa to beta-diversity, rare taxa were stepwise included into NMDS ordinations based on the Raup-Crick metric, which allows comparison of beta-diversity independent of changes in alpha-diversity (Chase *et al.*, 2011). The Raup-Crick metric gives the probability that sites have non-identical taxa composition. This probability was evaluated against 999 permutations of a community null model in which taxa are selected proportionally to their abundance.

CoNet, as implemented in Cytoscape (Faust *et al.*, 2012), was used to estimate co-occurrence relationships among OTUs (16S rDNA only). First, rare OTUs with fewer than five occurrences were removed. Pearson correlation, Spearman rank correlation, mutual information, as well as Bray–Curtis and Kullback–Leibler dissimilarities were calculated for ensemble network inference. From each of these five metrics, 1000 edges (positive and negative, respectively) with the strongest support (for example, largest correlation coefficients) were used for threshold selection. Associations with Benjamini–Hochberg false discovery rate *q*-values>0.05, inconclusive directionality or with support from less than two of the five metrics were removed. Spurious correlations were identified using randomization. Significant co-presence (positive co-occurrence) and mutual exclusion (negative co-occurrence) relationships between OTUs were displayed in a phylogenetic tree including all OTUs detected in the entire data set (Letunic and Bork, 2016). The standardized effect size (*z*-score) of the MNTD (mean nearest taxon distance) of the observed communities compared to a null model in which the tips of the phylogenetic tree were randomized (*n*=999) was used as a measure of phylogenetic clustering or overdispersion (function ses.mntd). The number of significant co-occurrence relationships between groups of bacteria was visualized using Cytoscape (Shannon *et al.*, 2003).

## Results

### Physico-chemical conditions

Turbidity ranged from 0.8 in IL7 to >60 NTU in the lakes directly receiving GrIS runoff (IL9 and IL15, Table 1). DOC concentrations varied fivefold (Table 1) and were paralleled by a strong gradient in DOM optical properties (Table 1). For example, the *S*_R_ parameter indicated that in lakes of low turbidity, DOM was dominated by low molecular weight compounds, whereas at high turbidity the opposite was true (Table 1). DOM fluorescence associated with proteins dominated the EEMs of all lakes. In total, Coble peaks b and t, which represent protein-like compounds (tyrosine and tryptophane) accounted for 87.1 to 92.8%, whereas peaks a, m and c reflecting humic-like compounds accounted for 7.2 to 12.9%. Despite large differences in nutrient concentrations, particularly in total phosphorus (maximum in IL15), chlorophyll-a concentrations were low (<1 μg l^−1^) in all systems.

### Community composition and diversity

**Figure 1:**
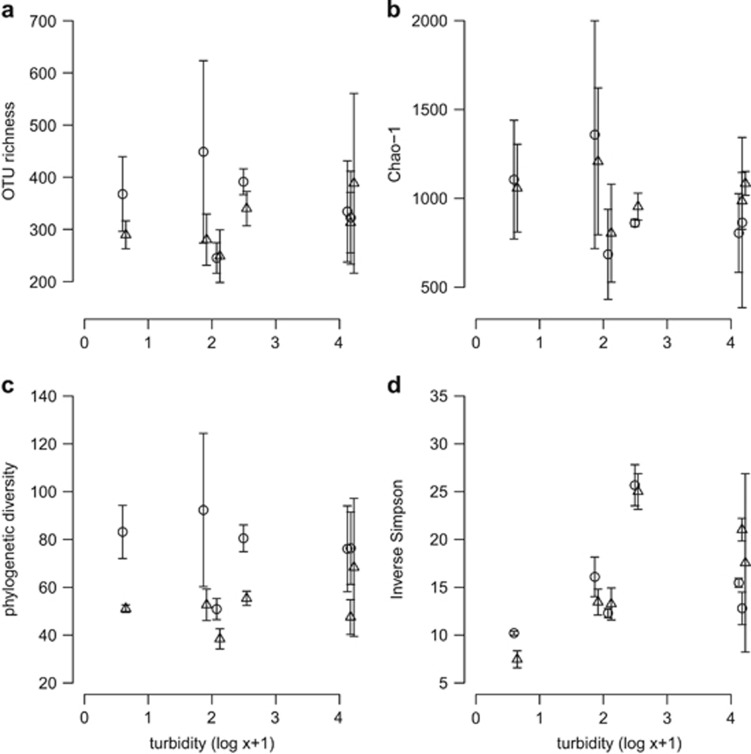
Alpha-diversity descriptors including (**a**) bootstrap OTU richness, (**b**) Chao-1, (**c**) Faith’s phylogenetic diversity index and (**d**) Inverse Simpson Index as a measure of evenness calculated from 16S rDNA (circles) and 16S rRNA (triangles) community fractions along the turbidity gradient. Shown are the average±s.d.s (*n*=3).

In total, 3859 and 3352 OTUs were recovered from rDNA and rRNA samples, respectively. Rarefaction curves for the different samples showed that not all diversity was sampled (Supplementary Figure S3). Between 245±25 and 449±175 OTUs were found in the rDNA and rRNA analyses for each lake, respectively. Abundant OTUs in the rDNA fraction tended also to be abundant in the rRNA fraction (Supplementary Figure S4a) and most taxonomic groups had a ratio of rRNA to rDNA of ∼1 (Supplementary Figure S4b). Actinobacteria were generally underrepresented in the rRNA fraction (mean rRNA:rDNA=0.64), whereas Alphaproteobacteria (mean rRNA:rDNA=3.40) and Deltaproteobacteria (mean rRNA:rDNA=2.86) were generally overrepresented in the rRNA fraction. No correlation between the number of OTUs, Chao-1 estimate and phylogenetic diversity with turbidity was found (Figure 1).

Bacterial community composition was significantly different among lakes (ANOSIM_DNA_ *R*=0.98, *P*<0.01; ANOSIM_RNA_ *R*=0.78, *P*<0.01) and non-metric multidimensional scaling indicated that community similarity was structured along the turbidity gradient (Supplementary Figure S5). Turbidity was the environmental variable that best explained the DNA-based community structure (*R*^2^=0.84), but temperature (*R*^2^=0.65) and TP (*R*^2^=0.56) were more strongly related to the RNA-based community structure than was turbidity (*R*^2^=0.55). Turbidity was positively related to lake area and total phosphorus and negatively related to water temperature and *S*_R_ (Supplementary Figure S6).

**Figure 2:**
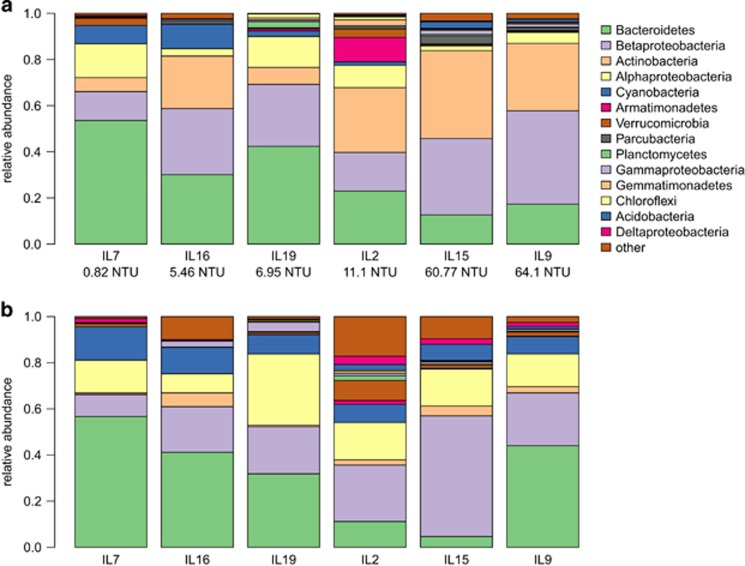
Community composition of the 16S rDNA (**a**) and 16S rRNA (**b**) fractions of the bacterioplankton communities along the turbidity gradient (lakes ordered by increasing turbidity). Bacteroidetes and Betaproteobacteria dominated the bulk and the active fraction of the communities with a shift in dominance between these groups along the turbidity gradient. Note that Actinobacteria were relatively more abundant in the rDNA fraction and less represented in the rRNA fractions. Alphaproteobacteria, in contrast, were relatively more abundant in the rRNA than in the rDNA fractions.

OTUs affiliated to Bacteroidetes, Betaproteobacteria and Actinobacteria were the most dominant in both the DNA and RNA fractions (Figure 2). While only a minor fraction of OTUs were shared among all lakes (DNA: 0.34%, RNA: 0.48%), OTUs that occurred in all lakes accounted for 23.2% of relative abundance in rDNA and 26.91% in relative abundance in rRNA. Bacteroidetes dominated in the clearest lake, but Betaproteobacteria and Actinobacteria were more abundant in lakes of intermediate and high turbidity. OTUs related to Acidobacteria Gp3, *Arcicella*, *Albidiferax*, Burkholderiales, Acidobacteria Gp6 and Methylophilaceae were exclusively found at the highest turbidity. In contrast, at the lowest turbidity, members of Cytophagaceae, Chitinophagaceae and Sphingobacteriales, and also *Flavobacterium*, *Limnohabitans* and *Cryobacterium* were found. Phytoplankton communities were represented mainly by Bacillariophyta (6972 sequences in 39 OTUs), Chlorophyceae (848 sequences in 26 OTUs) and Cryptophyceae (390 sequences in 24 OTUs).

### Rare versus abundant community members

Applying an arbitrary cutoff of 0.1% of relative abundance to differentiate between rare and abundant community members, we found between 47 and 81 abundant OTUs and between 478 and 1027 rare OTUs in the rDNA fractions of the different lakes. There were no significant relationships between the number of rare or abundant taxa and turbidity. Using the same cutoff, between 55 and 103 OTUs and between 485 and 946 OTUs were classified as abundant and rare in the rRNA fractions of these communities, respectively. Out of the 68 abundant OTUs found in the rDNA fraction of the most turbid lake, only eight OTUs were also classified as abundant in the clearest lake. In fact, the number of abundant OTUs shared between IL9, the most turbid system and the other lakes correlated well with turbidity (*R*^2^=0.97, *P*<0.01). Out of the 68 most abundant OTUs (rDNA) in the clearest lake, only 25% occurred also in the two most turbid lakes, and none had a relative abundance higher than 0.1%.

**Figure 3:**
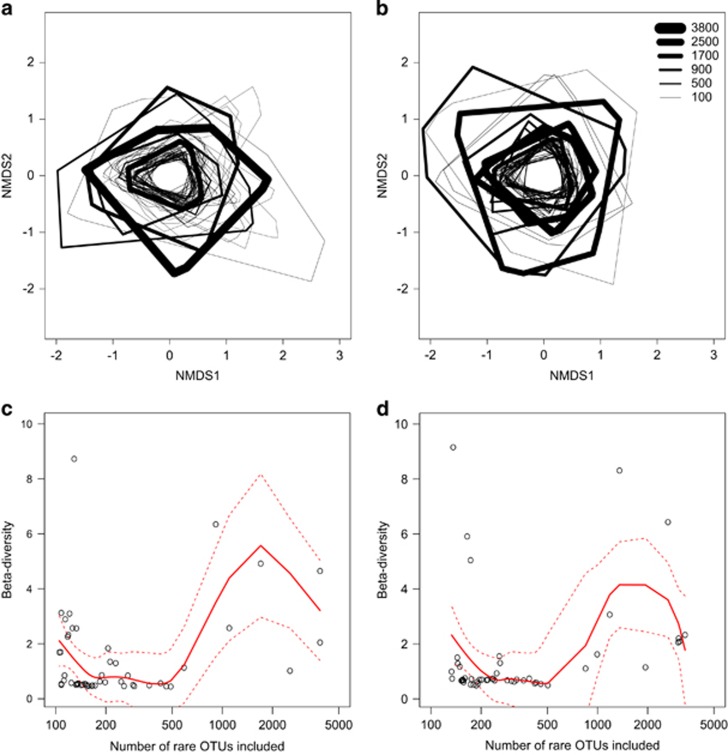
Non-metric multidimensional scaling ordinations (based on Raup-Crick metrics) for bulk (16S rDNA; (**a**) and active (16S rRNA; (**b**) fractions of the bacterioplankton communities. An increasing number of rare OTUs were included into the ordinations starting from the 100 most abundant OTUs and, in a stepwise manner, including rare taxa. The convex hulls include all samples such that the comprised area is a measure of beta-diversity. Line width reflects the number of OTUs included in the ordination. The bimodal distribution of beta-diversity (**c**, **d**) indicates that the 100–150 most abundant OTUs contributed to dissimilarity between samples. OTUs of intermediate rarity (for example, between 200 and 500 on the *x* axis) contributed to similarity among samples, whereas the inclusion of the rare biosphere again contributed to dissimilarity among the lake communities.

Using non-metric multidimensional scaling of community subsets and stepwise inclusion of rare OTUs, we found that rare and prevalent OTUs contributed to dissimilarity between sites (that is, beta-diversity) (Figure 3). This was similar for both rDNA and rRNA fractions. Quantification of the multivariate dispersion (measured as the area occupied by all samples in the ordinations) along a sequence of accumulating rarity showed that both rare and the most abundant OTUs contributed to similar extends to beta-diversity in these lakes (Figure 3). Inclusion of OTUs of intermediate abundance, on the other hand, reduced the multivariate spread of the samples, reflecting their shared occurrence in the lakes.

By drawing random subsamples from the species × site matrix (Supplementary Figure S7), we found that subsamples based on rDNA remained clearly separated among lakes, whereas this separation was less pronounced for the rRNA fraction. Although this analysis indicates that active community members tended to be shared among lakes, differences in the species-abundance distribution of bulk (rDNA) and active (rRNA) community members may also contribute to this patterns. Comparison of rank-abundance curves (Supplementary Figure S8) showed indeed that at high ranks, the relative abundance of rRNA-based OTUs was higher than for rDNA-based samples in all lakes.

### Phylogenetic structure of co-occurring taxa

**Figure 4:**
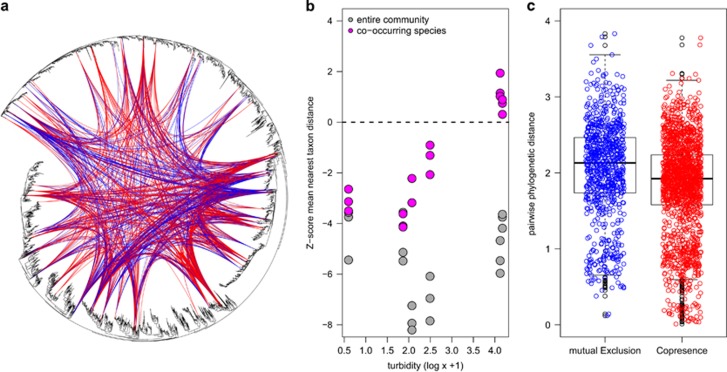
Circular view of a phylogenetic tree including all OTUs (**a**). Lines between OTUs represent significant positive (red) and negative co-occurrence relationships among these. Co-occurrence relationships are not evenly distributed across the phylogenetic tree but appears clustered. Comparisons of mean nearest taxon distance (MNTD) to null models for entire communities and the subset of OTUs involved in co-occurrence relationship are shown in panel **b**. Negative *z*-scores indicate phylogenetic clustering, whereas positive values indicate phylogenetic overdispersion. On average, phylogenetic distances between pairs of OTUs with positive co-occurrence relationships (Co-presence) were smaller than for pairs with negative co-occurrence relationships (mutual Exclusion) (**c**).

Ensemble network inference resulted in 2482 significant co-occurrence relationships among OTUs, out of which 1690 were positive (that is, co-presence) and 792 negative (that is, mutual exclusion) (Figure 4). In total, 326 OTUs were involved in significant associations, with members of Bacteroidetes (Sphingobacteria and Flavobacteria) featuring the highest number of co-occurrence relationships. For instance, OTU55, an unclassified member of Cytophagaceae, was involved in 105 significant co-occurrence relationships, whereas the median number of significant co-occurrence relationships per OTU was 10. Positive co-occurrence relationships dominated in all lakes (76.2±8.7%), but relatively more positive relationships were detected in clear lakes (Supplementary Figure S9). Most of the significant co-occurrence relationships were identified among OTUs classified as Bacteroidetes (*n*=232), followed by associations between Bacteroidetes and unclassified OTUs (*n*=115), Bacteroidetes and Actinobacteria (*n*=102), and between Bacteroidetes and Alphaproteobacteria (*n*=102) (Supplementary Figure S10). Co-occurrence relationships among phylogenetic groups other than Bacteroidetes were in general rare. For example, OTUs classified as Betaproteobacteria had 67 significant relationships with other betaproteobacterial OTUs and 34 relationships with OTUs classified as Alphaproteobacteria.

Co-occurrence relationships among OTUs were phylogenetically clustered (Figure 4). Visualization of the co-occurrence relationships on the phylogenetic tree revealed that several clades were not involved in co-occurrence relationships, whereas in other clades several members had significant co-occurrence relationships with other taxa (Figure 4a). Along the turbidity gradient, the communities were significantly phylogenetically clustered (MNTD *z*-scores<0, *P*<0.05, Figure 4b). The subsets of communities including only taxa involved in significant co-occurrence relationships were phylogenetically clustered at low turbidity (MNTD *z*-scores<0, *P*<0.05). Taxa with significant co-occurrence relationships in the most turbid lakes, in contrast, tended to be phylogenetically even or overdispersed (that is, similarly or more distantly related than expected by chance, MNTD *z*-scores≈0). Pairwise phylogenetic distance was significantly larger for pairs of OTUs involved in negative (mean pairwise phylogenetic distance: 2.04) than in positive (mean pairwise phylogenetic distance: 1.83) co-occurrence relationships (Figure 4c; Welch’s *t*-test, *P*<0.01).

## Discussion

Global climate change threatens the diversity adapted to the harsh environmental conditions of glacier-influenced lakes and streams (Jacobsen *et al.*, 2012; Wilhelm *et al.*, 2013; Peter and Sommaruga, 2016). On the other hand, glacier retreat leads to the formation of many new freshwater habitats in previously ice-covered areas (Slemmons *et al.*, 2013; Sommaruga, 2015). We did not find evidence for differences in alpha-diversity among lakes recently formed by the retreat of the GrIS (Figure 1), which contrasts with the finding for lakes influenced by the retreat of a small alpine glacier (Peter and Sommaruga, 2016). Abundant community members of the turbid lakes have also been detected in GrIS meltwaters (Cameron *et al.*, 2017) and different environments on, within and below the glacier may be sources of diversity to these newly formed turbid lakes. Unraveling the meta-community dynamics during early lake ontogeny remains, however, a future research task.

The bacterioplankton communities were structured by environmental factors such as high turbidity, low water temperature and by the concentration of nutrients and DOC (Supplementary Figure S5). The attenuation of light by suspended mineral particles (Rose *et al.*, 2014) is likely a key factor affecting the composition and abundance of phototrophic microbes in glacier-fed lakes. Given a reduced autochthonous production of organic matter, allochthonous carbon sources, most likely from sources on and within the glacier, may fuel secondary production. In fact, the optical signatures of the organic matter indicated the dominance of protein-like compounds, which prevail in glacier-derived carbon (for example, Hood *et al.*, 2009).

The role of the rare biosphere during the early ontogeny of lakes is currently not understood. We dissected the community structure of bulk and putatively active fractions of the bacterioplankton to test whether the rare biosphere accumulates or if rare taxa contribute to differences in community structure. The abundance of a bacterial population is determined by the balance of its growth rate and loss factors such as viral lysis and grazing. Whether a species is part of the ‘rare biosphere’ may therefore be linked to its past or present activity. A common assumption is that the rare biosphere is composed of mostly non-propagating members that assemble over time (Pedrós-Alió, 2006). However, at larger spatial scales, rare microorganisms exhibit biogeographic patterns (Lynch and Neufeld, 2015) and thus, they may contribute to beta-diversity. We found no evidence for such an accumulation of rare taxa. Investigating the multivariate spread of communities by sequentially including rare taxa (Figure 3), we show that OTUs of low abundance contributed substantially to beta-diversity among lakes formed by the retreat of the GrIS. This was the case for both the bulk and the putatively active fraction of the communities, suggesting that rare bacterioplankton community members respond actively to the oligotrophic conditions during the early phases of lake ontogeny. This supports the notion that rarity may be an evolutionary justified lifestyle adapted to low resource availability (Lynch and Neufeld, 2015; Newton and Shade, 2016). Similarly, Wilhelm *et al.* (2014) reported rare, but active taxa in glacier-fed stream biofilms and that a high turnover between active communities and inactive ‘seedbanks’ may reflect an adaptation to the harsh and fluctuating environmental conditions in glacier-influenced ecosystems. To further understand how rare and prevalent taxa contribute to dissimilarity of the rDNA and rRNA fractions, we used randomly subsampled non-metric multidimensional scaling ordinations (Supplementary Figure S7). Random subsampling of a community maintains the abundance structure, and more abundant OTUs are more likely to be included. This analysis showed that the rDNA communities were clearly separated among lakes. Randomization of the rRNA community fraction, however, reduced dissimilarity between them. These results suggest that the active fractions of the communities are rather shared among the lakes.

A ‘seeding’ effect of rare species has been suggested to influence the assembly of communities (that is, rare but early arriving taxa may occupy niches, thus inhibiting invasions) (Jousset *et al.*, 2017). Assuming that the turbidity gradient reflects the sequence of community assembly in these lakes, we tested whether the number of rare taxa in the turbid lakes correlates with the number of taxa in the clear lakes. Although we found a large turnover in the number of abundant taxa along the turbidity gradient and that most abundant taxa in the clear lake were not present in the turbid lakes, we did not find evidence for such a ‘seeding’ effect of rare OTUs. However, the identity of rare taxa and knowledge of the functional overlap with potentially invading taxa may be critical to elucidate such an effect during early lake ontogeny.

We further assessed the role of co-occurrence relationships during early lake ontogeny. Co-occurrence among taxa may arise from shared environmental preferences or may be the result of positive or negative interactions such as facilitation or competition and apparent interactions such as shared pathogens (Fuhrman and Steele, 2008). Here, we show that the relative distribution of positive and negative co-occurrence relationships changes along the turbidity gradient (Supplementary Figure S10). Further, although the differences were small, taxa involved in positive co-occurrence relationships were more closely related than taxa involved in negative relationships (Figure 4c). This may indicate that competitive interactions could be important during the colonization of these oligotrophic habitats when predation pressure is low due to a truncated food web (Sommaruga, 2015). However, as lakes loose connectivity with the glacier and become less turbid, positive interactions even among closely related community members may drive the community structure. On the other hand, a massive import of cells with the glacier meltwater could also result in negative co-occurrence relationships with the resident lake microbiota, irrespective of interactions among species or habitat preferences. The average cell numbers exported from the GrIS are rather low (for example, 8.3 × 10^4^ cells ml^−1^; Cameron *et al.*, 2017). Hence, we argue that such mass effects would likely not play a very important role. However, large amounts of meltwaters during warm periods in relation to the residence time of the lakes need to be considered.

Under the assumption that phylogenetic relatedness reflects species niche similarity, phylogenetic clustering or overdispersion may result from environmental filtering or competition, respectively (Mouquet *et al.*, 2012). Compared with the null expectation of a random phylogenetic structure, the bacterioplankton communities were consistently clustered (Figure 4). This reflects a scenario in which closely related taxa with similar niche preferences dominate the communities. Furthermore, evaluation of the subsets of the communities that were involved in positive or negative co-occurrence relationships indicated shifts from overdispersion or evenness to phylogenetic clustering during the transition from turbid to clear lake states. Taken together, this shows that groups of phylogenetically related taxa drive bacterial community structure during early lake ontogeny, with a relatively higher number of negative associations among taxa in highly turbid lakes.

In conclusion, the strong environmental gradients along the sequence from glacier-fed, turbid to clear lake states and the truncated food web of these newly formed lakes offered ideal opportunities to elucidate how bacterioplankton communities are structured at the onset of lake ontogeny. We show that environmental factors related to glacier retreat provide potentially transient niches and that rapid successional changes take place during the transition from turbid to clear states. Rare but active community members contribute to dissimilarity between lakes, which contradicts the notion of an accumulation of the rare biosphere during lake ontogeny. Competitive interactions among phylogenetically closely related taxa may be a strong driver of community composition in the most turbid lakes. When these lakes become less turbid, relatively more positive interactions occur, also among phylogenetically more closely related taxa. Future work may attempt to link community structure to the potential local and regional sources of microbial diversity to clarify the role of dispersal of rare, abundant, active or inactive bacterial cells.

## Data availability

The data sets generated during the current study are available in fighsare under https://doi.org/10.6084/m9.figshare.c.3855733.v1. Raw sequence data have been deposited in the Sequence Read Archive under Accession numbers SAMN07514214 and SAMN07514215.

## Figures and Tables

**Table 1:** Lake main characteristics and environmental background data

*Lake ID*	*Area (ha)*	*Max depth (m)*	*Temp. (*°*C)*	*pH*	*Cond. (μS cm*^*−1*^)	*Turb. (NTU)*	*TP (μg l*^*−1*^)	*TN (mg l*^*−1*^)	*Chl-a (μg ^l^*^*−1*^)	*BA (10*^*5*^ *cells ml*^*−1*^)	*DOC (mg l*^*−1*^)	*S_R_*	*Coble peaks (%) b,t,a,m,c*
IL2	5.76	12.5	10.2	7.89	119.9	11.1	10	0.09	0.60	32.5	1.07	2.15	75.8, 21.2,6.7,2.9,1.9
IL7	0.21	5.3	12.1	8.29	252.4	0.8	6	0.42	0.72	NA	5.61	1.84	67.3, 21.2,6.7,2.8,1.9
IL 9	126	9.2	4.9	8.37	–	64.1	23	0.11	0.86	NA	2.22	1.60	70.8, 19.9, 4.7, 3.5, 1.2
IL 15	247	36.0	0.7	7.73	14.0	60.1	64	0.04	0.12	3.01	1.82	1.37	64.7, 22.4, 7.1, 3.5, 2.4
IL16	0.05	2.0	9.1	8.07	250.2	5.5	6	0.19	0.36	3.72	3.09	2.33	75.4, 17.4, 3.6, 2.2, 1.4
IL19	1.11	7.0	9.1	7.77	107.0	6.9	8	0.09	0.48	2.36	4.36	2.17	70.9, 21.5, 3.8, 2.5, 1.3

Abbreviations: BA, bacterial abundance; Chl-a, chlorophyll-a; cond., specific conductivity; DOC, dissolved organic carbon concentration; NA, not analyzed; *S*_R_, absorbance slope ratio; Temp., surface water temperature; TN; total nitrogen; TP, total phosphorus; turb., turbidity. The relative distribution of fluorescence among of Coble peaks is shown for the protein-like peaks b and t, and the humic-like Coble peaks a, m and c.
