# Supplementary material for: Changes in bacterioplankton community structure during early lake ontogeny resulting from the retreat of the Greenland Ice Sheet
Source: ISME J. 2017 Oct 31;12(2):544–55. doi: 10.1038/ismej.2017.191 (PMC5776470; doi:10.1038/ismej.2017.191)
Supplement: Supplementary Figures [file ismej2017191x1.docx]

**Supplementary Figure S1** Map of the study area indicating the sampled lakes


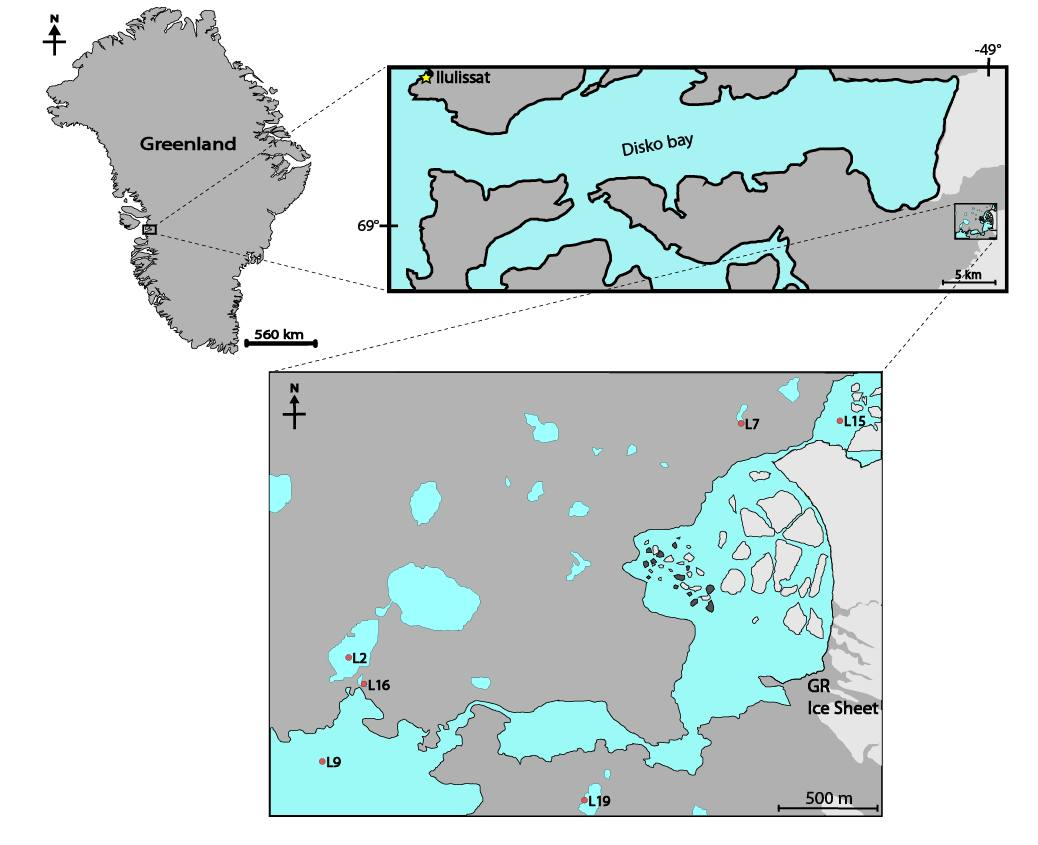


**Supplementary Figure** **S2** Landsat images from 1972 to 2008 showing the approximate time of formation of the lakes in this area. The year and day of the year (DOY) is given. Source: EarthExplorer (USGS, https://earthexplorer.usgs.gov/) or Google Earth pro. Aerial orthoimage from 1985 was provided by Anders A. Bjørk.

1972 (DOY: 279)


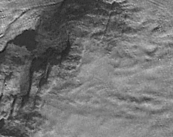


1985 (DOY: 190)


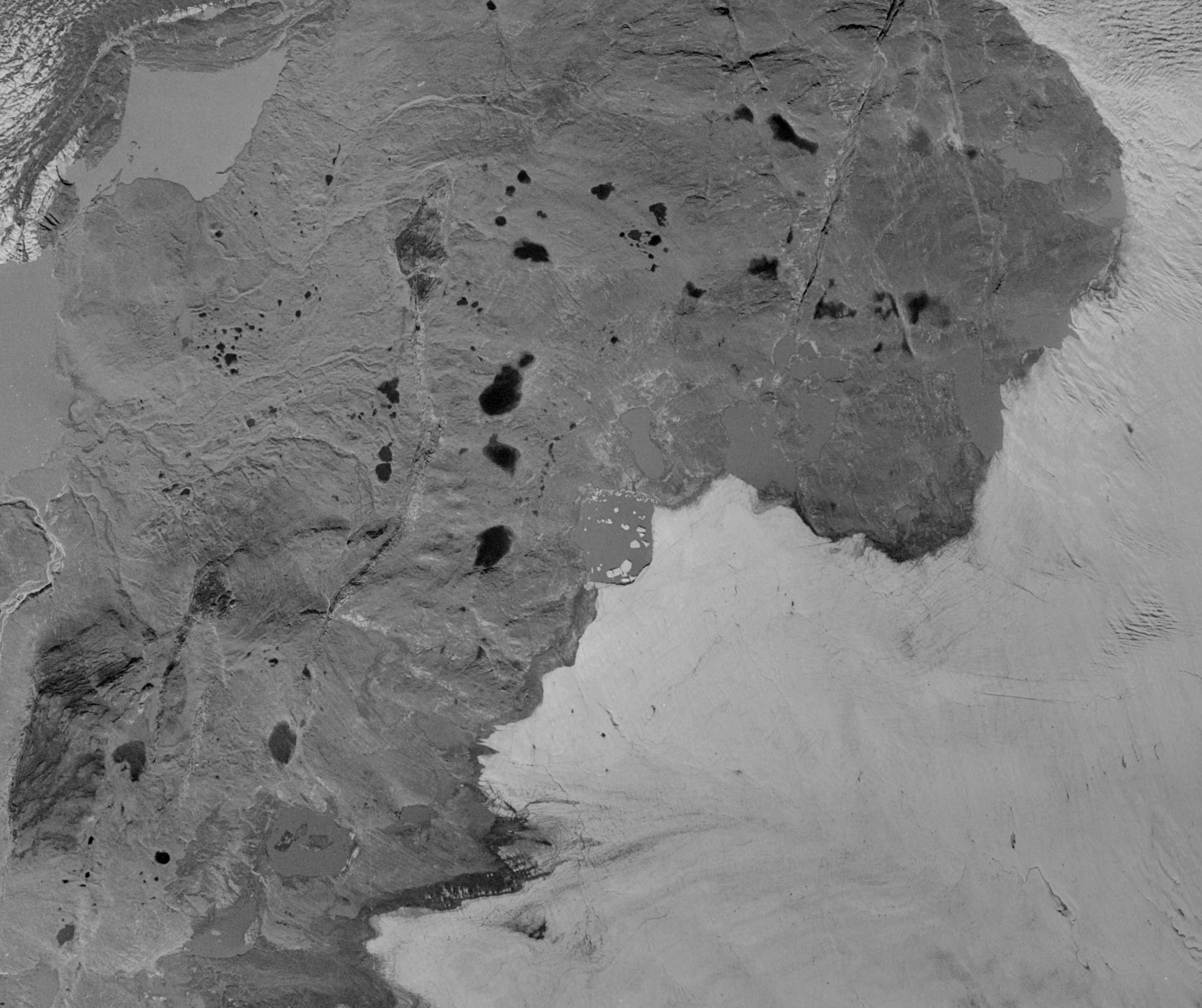


L2

1987 (DOY: 206)


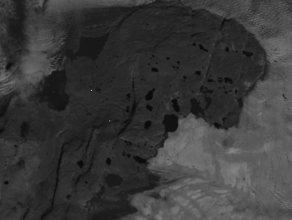


L2

1994 (DOY: 193)


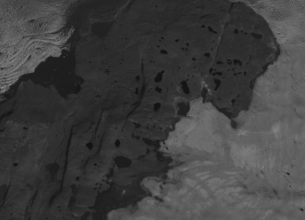


L2

1998 (DOY: 165)


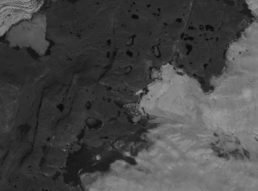


L2

L7

2002 (DOY: 246)


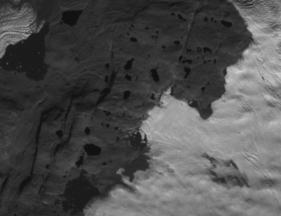


2008 (DOY 228)


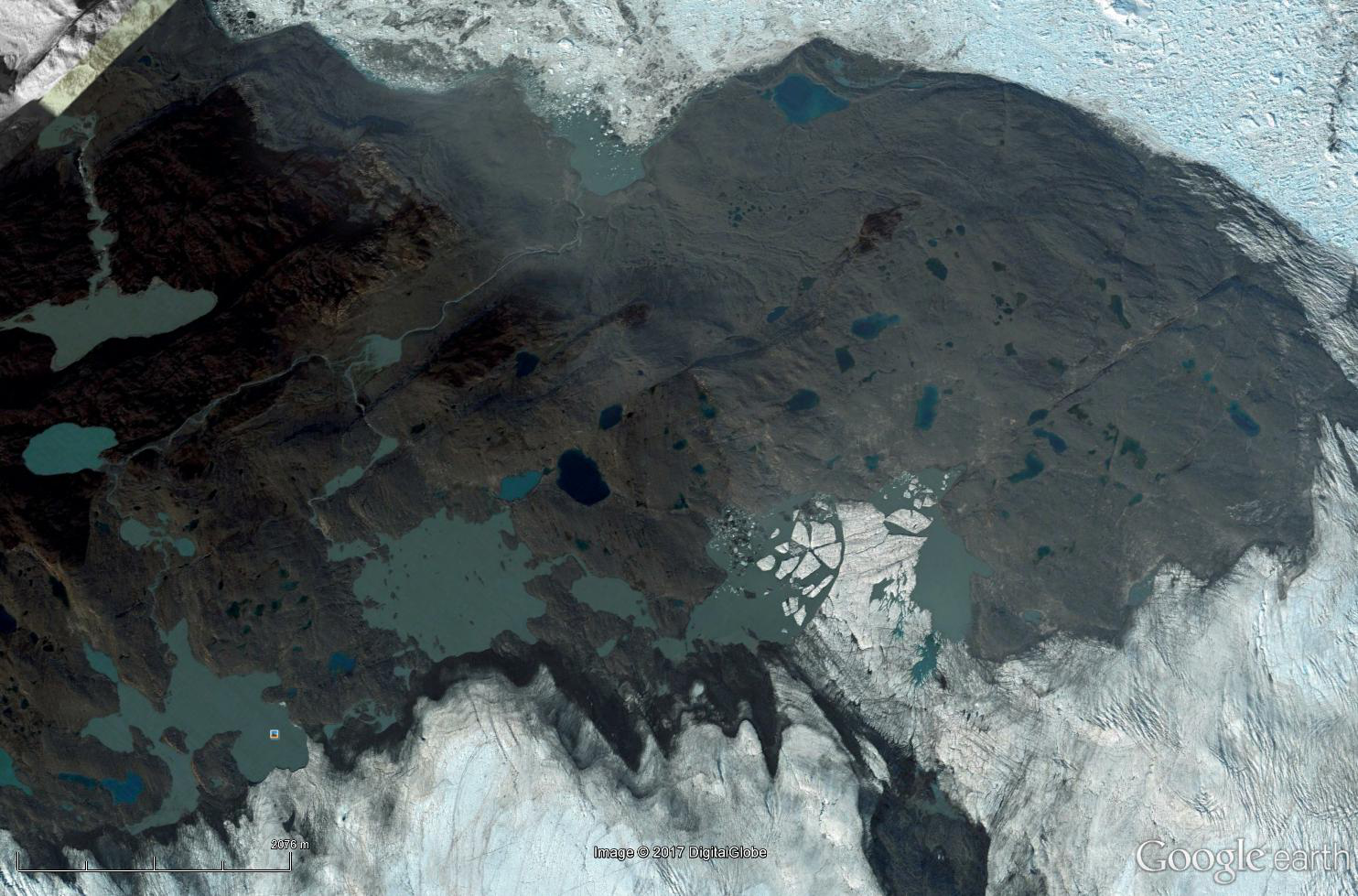


L16

L15

L19

L7

L2

L9


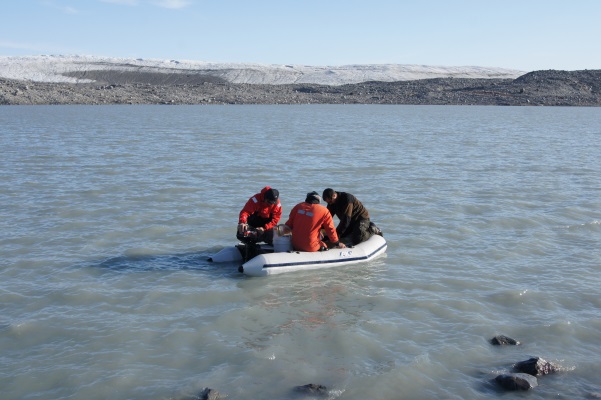

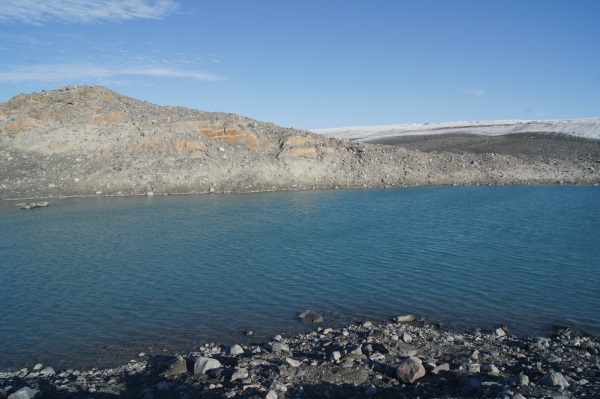


**L19**

**L9**


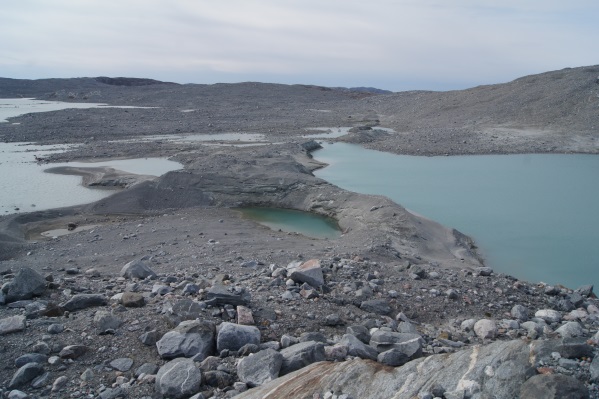

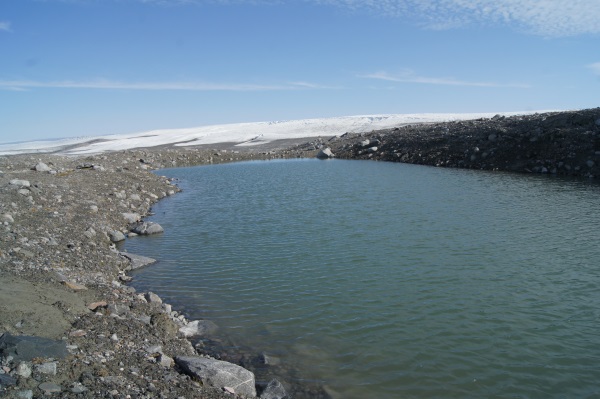


**L16**

**L7**

**L2**


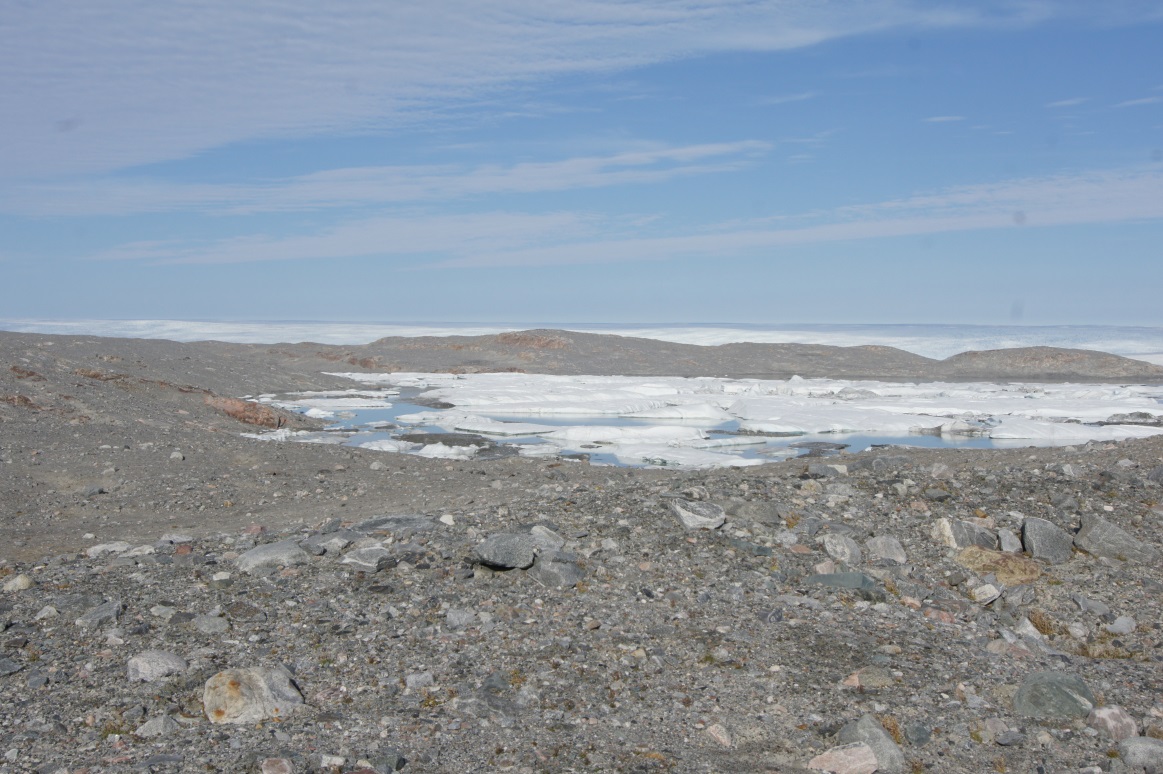


**L15**

Images of the lakes at the time of sampling in 2012.

**Supplementary Figure S3** Rarefaction curves showing the number of OTUs as a function of the number of sequences for the replicated (n=3) rDNA (a) and rRNA (b) samples. While the bulk (rDNA) diversity was considerably well sampled, the diversity of the active fraction of the community (rRNA) appears to be undersampled.

**b**

**a**

**Supplementary Figure S4** For each of the six lakes, read counts for OTUs in rRNA and rDNA are shown (**a**). For this, the rRNA and rDNA sequence files were merged after quality trimming and clustered into OTUs. The red dotted line represents the 1:1 line, whereas the three black lines reflect linear regressions for each of the three independent replicates. Boxplots of the ratios of 16S rRNA to 16S rDNA (OTUs level) are shown for major taxonomic groups (**b**). While most groups had a median ratio close to 1, Actinobacteria (n=15) were underrepresented in the rRNA fraction, whereas Deltaproteobacteria (n=14) were overrepresented in the rRNA fraction.

**IL 9**

**IL 7**

**IL 2**

**IL 19**

**IL 16**

**IL 15**

**a**

**b**

**Supplementary Figure S5** Non-metric multidimensional scaling ordination showing environmental factors driving dissimilarities of the bulk (rDNA, **a**), the active fraction of the bacterial communities (rRNA, **b**) and the phytoplankton communities (**c**) among the sampled lakes. The table provides adjusted R^2^ and P values as outputted from envfit. Note that turbidity is strongly correlated to all community structures (R^2^ _DNA_ = 0.84, R^2^ _RNA_ = 0.55, R^2^ _phytoplankton_ = 0.70, p< 0.01). For the active fraction of the community (RNA, panel **b**), temperature (R^2^ = 0.65, p<0.01) and TP (R^2^ = 0.56, p<0.01) explained more of the community structure than turbidity, while for the phytoplankton communities the absorbance slope ratio (*S_R_*) was more strongly related to community composition than turbidity (R^2^ = 0.80, p<0.01).

**c**

**b**

**a**

|  | DNA | | RNA | | phytoplankton | |
| --- | --- | --- | --- | --- | --- | --- |
|  | R^2^ | P | R^2^ | P | R^2^ | P |
| turbidity | 0.84 | 0.001 | 0.55 | 0.008 | 0.70 | 0.001 |
| area | 0.53 | 0.003 | 0.25 | 0.139 | 0.46 | 0.014 |
| temperature | 0.78 | 0.001 | 0.65 | 0.002 | 0.51 | 0.007 |
| pH | 0.17 | 0.22 | 0.55 | 0.004 | 0.42 | 0.011 |
| conductivity | 0.26 | 0.103 | 0.14 | 0.334 | 0.20 | 0.231 |
| TP | 0.47 | 0.006 | 0.56 | 0.002 | 0.55 | 0.004 |
| TN | 0.65 | 0.002 | 0.60 | 0.001 | 0.35 | 0.049 |
| DOC | 0.69 | 0.001 | 0.55 | 0.005 | 0.26 | 0.144 |
| *S_R_* | 0.42 | 0.014 | 0.30 | 0.097 | 0.80 | 0.001 |
| Coble peak b | 0.35 | 0.05 | 0.44 | 0.016 | 0.23 | 0.178 |
| Coble peak t | 0.29 | 0.08 | 0.50 | 0.009 | 0.32 | 0.073 |
| Coble peak a | 0.28 | 0.08 | 0.49 | 0.006 | 0.67 | 0.001 |
| Coble peak m | 0.35 | 0.04 | 0.41 | 0.022 | 0.44 | 0.026 |
| Coble peak c | 0.43 | 0.02 | 0.24 | 0.129 | 0.20 | 0.216 |

**Supplementary Figure S6** Scatterplot matrix showing correlations between key environmental variables (**a**) and turbidity and DOM related parameter (**b**). The diagonals contain histograms and the variable names, below the diagonal pairwise comparisons are plotted with linear models shown. Pearson correlation coefficients are given above the diagonal with font size scaled respectively. Turbidity is positively related with lake area, conductivity and TP and negatively correlated to temperature, TN, DOC concentration and absorbance slope ratio (*SR*). Coble peaks b, t, a, m and c are abbreviated with the respective letters.

**a**

**b**

**Supplementary Figure S7** NMDS ordinations based on Bray-Curtis similarity of randomly subsampled communities each composed of half of the reads detected in rDNA (a) and rRNA samples (b), respectively. Each point represents a randomized fraction of the original communities (color coded) which contained between 1863 and 6201 reads for rDNA samples and between 1052 and 10466 for rRNA samples. The likelihood of being included into a subsampled community depends on the initial relative abundance of an OTU, with more abundant OTUs being more likely to be included in the analyses. Therefore, this approach allows to visualize the effects of abundance distribution to similarities between sites. If numerous abundant community members are shared among sites, subsampled communities will appear more closely in the ordination. Note the separation between lakes in the bulk communities and the large spread among lakes in the active fraction of the communities, reflecting the effects of different rank-abundance distributions of bulk and active fractions of the communities on similarity.

**Supplementary Figure S8** Comparison of normalized rank-abundance distributions between rDNA (dark colors) and rRNA (light colors) community fractions.

**Supplementary Figure S9** Progression of the relative distribution of co-occurrence relationships along the turbidity gradient.

**Supplementary Figure S10** Summary of the number of significant co-occurrence relationships among broad phylogenetic groups. The thickness of the line connecting two groups reflects the number of significant co-occurrence relationships. Note that many relationships were detected between members of the same phylogenetic group (loops).
